# Assessment of Unstimulated Whole Salivary Tumor Necrosis Factor Alpha (TNF-α) and Cellular Micronuclei Levels in Snuff (Naswar) Users and Non-Users for Early Diagnosis of Oral Squamous Cell Carcinoma

**DOI:** 10.3390/ijerph18147230

**Published:** 2021-07-06

**Authors:** Waqar Muhammad, Muhammad M. Khan, Shafaq Zafar, Montaser N. Alqutub, Abdulrahman M. AlMubarak, Sameer Mokeem, Zafar A. Khan, Muhammad K. Usman, Naseer Ahmed, Nada Aldahiyan, Fahim Vohra, Tariq Abduljabbar

**Affiliations:** 1 Department of Oral Pathology, Peshawar Medical College, Warsak Road, Peshawar 25160, Pakistan; waqar_jadoon08@yahoo.com (W.M.); mumtazkhan@yahoo.com (M.M.K.); dr.khalid78@yahoo.com (M.K.U.); 2 Department of Oral Pathology, Riphah International University, Islamabad 46000, Pakistan; 3 Department of Pharmacology, Peshawar Medical College, Warsak Road, Peshawar 25160, Pakistan; dr.shafaqzafar@gmail.com; 4 Department of Pharmacology, Riphah International University, Islamabad 46000, Pakistan; 5 Department of Periodontics and Community Dentistry, College of Dentistry, King Saud University, Riyadh 11545, Saudi Arabia; Alqutub@hotmail.com (M.N.A.); amalmubarak@ksu.edu.sa (A.M.A.); smokeem@ksu.edu.sa (S.M.); 6 Department of Oral & Maxillofacial Surgery, College of Dentistry, Jouf University, Sakaka 72311, Saudi Arabia; dr.zafar.khan@jodent.org; 7 Department of Prosthodontics, Altamash Institute of Dental Medicine, Karachi 75500, Pakistan; naprosthodontist@gmail.com; 8 Department of Restorative Dental Science, College of Dentistry, King Saud University, Riyadh 11545, Saudi Arabia; naldahiyan@ksu.edu.sa; 9 Department of Prosthetic Dental Science, College of Dentistry, King Saud University, Riyadh 11545, Saudi Arabia; fvohra@ksu.edu.sa

**Keywords:** micronuclei, TNF-α, snuff dippers, naswar, smokeless tobacco, oral cancer

## Abstract

The aim of the study was to investigate the unstimulated whole saliva (UWS) tumor necrosis factor alpha (TNF-α) and cellular micronuclei in snuff dippers (Naswar) compared to healthy control subjects. The case control study was conducted over 9 months at a tertiary care center. Sixty patients were divided into two groups: Snuff dippers (SD) (Naswar) and non-snuff dippers (NSD) (control subjects). The included self-reported SD used Snuff twice daily for more than 12 months. UWS was collected and salivary TNF-α assessment was performed using enzyme-linked immunosorbent assay (ELISA). For cellular micronuclei, buccal mucosa was brushed to obtain cells in Naswar users, fixed with a dibutylphthalate polystyrene xylene (DPX) mounting to view micronuclei. Means and standard deviations were compared using the t-test and outcomes were related using Pearson correlation, considering *p* ≤ 0.05 as significant. The mean age of participants was 38.85 ± 11.56 years. The mean duration of snuff use was 20.43 ± 12.79 years and the common site for Naswar placement was the lower vestibule (*n* = 19, 63.3%). TNF-α levels among SD were 9.6 ± 3.3 pg/mL, which were significantly higher than levels in NSD, 5.2 ± 3 pg/mL (*p* < 0.05). The number of cellular micronuclei in SD was 30.7 ± 7.8, which was comparatively higher than in NSD, which was 9.2 ± 3.3 (*p* < 0.05). The duration of snuff use was positively correlated to TNF-α levels (*p* = 0.048) rather than the micronuclei number (*p* = 0.97). SD showed higher levels of TNF-α and cellular micronuclei compared with NSD (control subjects); a positive correlation was shown with the duration of snuff use. We conclude that TNF-α and micronuclei are potential salivary biomarkers for an oral biological effect in snuff (Naswar) users.

## 1. Introduction

Oral cancer includes lesions of the oral cavity, oropharynx, and salivary glands. Oral squamous cell carcinoma (OSSC) is the most frequent oral cancer, comprising more than 90% of all oral cancers [1]. The morbidity and mortality rates of OSCC in males are higher (6.6 and 3.1 per 100,000, respectively) compared with females (2.9 and 1.4 per 100,000, respectively) [2]. The global burden of lip and buccal vestibule cancers is widely increasing among developing countries due to the use of tobacco among elderly men [3,4]. The transformation of potential premalignant lesions is often triggered by various carcinogenic sources. These include tobacco, betel nut, and alcohol, which induces genotoxic effects and causes the exfoliation of oral cells [4]. However, early identification of mucosal changes facilitates early recognition and effective implementation of preventive measures. Unfortunately, despite an early identification of mucosal changes, there remains a 50% chance for recurrence [5]. Thus, early biomarker detection in high risk patients is the key to reduce oral cancer incidence and improve survival rate.

Naswar is a niche powdered snuff, frequently used in South and Central Asia regions and countries including Pakistan, India, and Sri Lanka [6]. Frequent use and continuous snuff-dipping deposit prodigious tobacco content into the tissues and stains the teeth. Pérez-Ortuño et al. [7] reported that nitrosamine is the main component of tobacco, which has a potent mutagenic effect on chromosomes and induces inflammatory reactions. Nicotine absorption per dose is higher with chewing tobacco (approximately 4.5 mg) and snuff (approximately 3.6 mg) in comparison to the amount absorbed from smoking tobacco (approximately 1.0 mg) [8]. Prolonged use of betel nut and tobacco in snuff dippers leads to slow chemical release, which, combined with calcium lime, enhances the reactive oxygen species production and damages cellular (Deoxyribonucleic acid) DNA [7]. Subsequently, the resultant chromosomal aberration occurring during the anaphase stage produces micronuclei, round-oval extranuclear cytoplasmic bodies and deranged DNA cells [9].

The literature has stated that a high level of salivary tumor necrosis factor alpha (TNF-α) acts as an early indicator for the diagnosis of OSCC in contrast to histopathological review [8]. TNF-α is an inflammatory mediator mostly induced in chronic inflammatory reactions, mainly in response to foreign particles [10]. Thus, the deposits of smokeless tobacco in tissues have been identified as a risk factor for inducing overexpression of TNF-α. The extended use of smokeless tobacco deranges the gene expression of many interleukins and TNF-α; hence, increasing the vulnerability of the high-risk patient [11]. In addition, micronucleus assay is considered an effective technique to evaluate the cytological changes due to carcinogenic elements [12]. It can detect mitotic interference or breakage of chromosomes that are associated with cancer development [13,14]. It is suggested that the presence of micronuclei in exfoliated buccal cells shows genotoxic changes among the dividing cells of the basal layer. Moreover, this technique is noninvasive and sampling can be done repeatedly from the same site [15]. Thus, biomarkers such as TNF-α and cellular micronuclei can be used as good prognostic indicators for early diagnosis of OSSC [16].

Regardless of recent advances, the diagnosis of OSCC is often reported in the late stages of cancer, which reduces the survival rate and compromises prognosis [10]. Thus, salivary biomarkers are appreciated as an effective diagnostic tool for the early identification and prediction of cancer. However, due to the lack of recognition of Naswar as a carcinogen by the International Agency for Research on Cancer (IARC), limited research is conducted regarding the efficacy of salivary biomarkers in snuff dippers [17,18]. Thus, the present study aimed to investigate the TNF-α levels and the cellular micronuclei number in snuff dippers (SD) compared with non-snuff dippers (NSD) (healthy control subjects).

## 2. Materials and Methods

### 2.1. Ethical Consideration

The protocol for the study was designed under the standards of the Helsinki declaration (1964) and approved by the Prime Foundation institutional review board, Peshawar, Pakistan (Prime/IRB/2020-258). Subsequent to the approval of the protocol, the participants were presented with informed consent for their voluntary participation. Each patient was given the right to withdraw without any consequences. The study was reported using the STROBE guideline (Appendix A).

### 2.2. Study Design and Participants

The analytical case control study was conducted over a period of 9 months in 2019 at the outpatient department of a tertiary care institute. A total of 60 patients, age ranging from 18 to 62 years without any history of oral cancer was recruited to participate in the study. The patients were divided into two groups based on the history of snuff dippers (Naswar) and non-snuff dippers (NSD) (control subjects).

### 2.3. Inclusion and Exclusion Criteria 

After noting previous abuse history, medical status, and clinical examination, patients were categorized into groups based on snuff dipping. Self-reported SD were using snuff (Naswar) twice daily for more than 12 months without any mucosal lesions and systematic disease. The self-reported SD group participants had an intact oral mucosa i.e., without premalignant and frank malignant lesions. Self-reported NSD were individuals who did not habitually use snuff in the last 12 months (control subjects). Excluded individuals included patients with a history of abuse except for snuff, chronic periodontitis, alcoholics, workers associated with occupational hazards, and those with incomplete consent forms. In addition, patients with a history of oral lesions, pathological dry mouth syndrome or medications such as antihistamines, antihypertensives, anticholinergics, antidepressants, and bronchodilators were also excluded from the study due to a hindrance in the ability to collect sufficient unstimulated saliva samples.

### 2.4. Questionnaire

All included subjects completed a questionnaire to gather critical information prior to clinical examination. The questionnaire included information to be completed by patients self-reporting on their age, gender, abuse history (duration of snuff use and the site of snuff placement), and medical history, including drug use. Furthermore, an experienced senior clinical consultant (W.M.) undertook an oral examination, including assessment of oral hygiene and periodontal screening using the community periodontal index (CPI) (WHO standard) to assess the presence or absence of gingival bleeding, calculus, or deep pockets [19]. The oral cavity is divided into sextants and using a CPITN probe, an index was determined.: Code 0—healthy periodontium, Code 1—bleeding on gentle probing, Code 2—calculus deposits on probing, Code 3—pocket 4-5 mm, Code 4—pocket 6mm or more. The teeth examined were 17, 16, 11, 26, 27, 37, 36, 31, 46, and 47. Each patient was provided with oral hygiene instructions at clinical evaluation.

### 2.5. Unstimulated Whole Saliva (UWS) Collection

Patients were requested to perform a mouth rinse (3 min) with distilled water before proceeding with the saliva and epithelial cells collection from 10:00 a.m. to 12:00 p.m. After 10 min, the patient’s saliva (2‒3 mL) was collected in a sterile Falcon tube of 5 mL, under non-stimulatory conditions. The process included swallowing first, followed by a forward head tilt, and spitting out all UWS into the sterile tube through a plastic funnel. The collected UWS was centrifuged at 4000× *g* for 10 min to filter debris. Saliva was transported to the pathology laboratory and stored at −80 °C.

### 2.6. Enzyme-Linked Immunosorbent Assay (ELISA)

The TNF-α assay was performed using the DeQuantoTM Human TNF-α ELISA kit, (# QT 4001) according to the manufacturer’s instructions. The ELISA technique used in the study was the sandwich method. After thawing the tube for each sample, two 0.5 mL aliquots were made that were stored at −30 °C for the ELISA assay. All reagents were maintained at room temperature with microplate preheated for 15 min (96-well microplates). To prepare a 750 mL solution, a 30 mL concentrated wash buffer was prepared with 720 mL distilled water. The prepared solution was centrifuged at 10,000× *g* for a minute followed by preparation of working solution at 500 pg/mL using 1 mL standard and sample diluents for reference and left for 10 min. Dilutions were made in 500, 250, 125, 62.5, 31.25, 15.63, 7.81, 0 pg/mL sequence. Biotinylated diluents (Elabscience, Houston, TX, USA, E-EL-H0109) were used to prepare the concentrated solution of 1x from a 100x diluted solution. Similarly, a 100x concentrated HRP conjugate working solution was diluted to 1x concentration with its diluents.

Well plates were incubated at 37 °C for 90 min. After filtering the liquid, a biotinylated detection antibody at 100 µL was added and incubated for 1 h at 37 °C. Subsequently, the solution aspired was washed thrice and added to the 100 µL HRP conjugate (Elabscience, Houston, TX, USA), followed by incubation at 37 °C for 30 min. An amount of 90 µL of substrate reagent (Elabscience, Houston, TX, USA) was added and incubated for 15 min at 37 °C. The plate’s outcome was read at 450 nm and calculated following the addition of stop solution (50 µL of 0.16 M sulfuric acid).

### 2.7. Cellular Micronuclei Assessment

For cellular analysis, the buccal mucosa was brushed 10 times using a sterile cytobrush (Histobrush, Hardwood Products Company, Guilfor, ME, USA) to obtain cells from buccal vestibule in SD and NSD subjects. Each dipping site of Naswar was brushed to compare the obtained cells with the control subjects. The obtained specimen was spread on the prepared glass and fixed with 80% methanol after drying for 20 min; 3 slides were prepared per patient. Subsequently, Giemsa staining was performed with a 10% solution for 20 min. After washing and drying, the DPX mounting medium (Merck KGaA, Darmstadt, Germany) was poured and pressed on the slide, followed by storage at room temperature for microscopic analysis (Binocular NSL CX23, Olympus, Japan). The cytological smears were observed microscopically with a consultant oral histopathologist (A.M.). The slides were viewed under oil immersion lens at 1000x magnification to identify and then count the micronuclei per cell. Careful counting existed to avoid overlapping of fields and repeated counting of same cells. A number of 1000 cells were counted for each case and Tolbert et al. criterion was followed for identification and scoring of micronuclei [20].

### 2.8. Data Analysis

Data were analyzed using a statistical package for social sciences (SPSS version 23, Chicago, IL, USA). For categorical variables, frequencies and percentages were computed. Quantitative variables were measured and compared using means and standard deviations. An independent sample t-test was applied to compare the mean difference among the study groups in terms of TNF-α and micronuclei. Pearson correlation was employed to compare the difference between snuff and non-snuff users regarding age and duration of snuff use and considering *p* ≤ 0.05 as significant.

## 3. Results 

### 3.1. General Characteristics of the Participants

All sixty participants recruited in the study were men with a mean age of 38.85 (± 11.56) years, divided into two groups (*n* = 30), SD and NSD (Table 1). The age range in each group was SD (41.9 ± 12.2) and NSD (35.8 ± 10.4). The mean duration of snuff use was 20.43 years (± 12.79) and the most commonly used dipping site was the lower vestibule (*n* = 19, 63.3%). The upper vestibule was the site for snuff dipping in 36.6% of subjects (*n* = 11). A number of 8 (26.6%) snuff users showed poor oral hygiene and 22 (73.3%) had fair oral hygiene. Conversely, 18 control subjects (60%) had fair oral hygiene and 12 control subjects (40%) had relatively good hygiene. Overall, good, fair, and poor oral hygiene was observed in 20% (*n* = 12), 66.6% (*n* = 40), and 13.3% (*n* = 8) of study subjects (Table 1), respectively. The average bleeding sites among SD and NSD were 8.7 % (0.0–19.6) and 5.5 % (0.0–13.0), respectively, (*p* = 0.071). The presence of calculus between SD and NSD was 12.6% and 8.8%, respectively, (*p* = 0.135). Finally, the CPI index scores among SD and NSD were 2.13 (0–4) and 1.75 (0–4), respectively, (*p* = 0.444).

### 3.2. Assessment of TNF-α and Micronuclei Cells in the Study Groups

**Figure 1:**
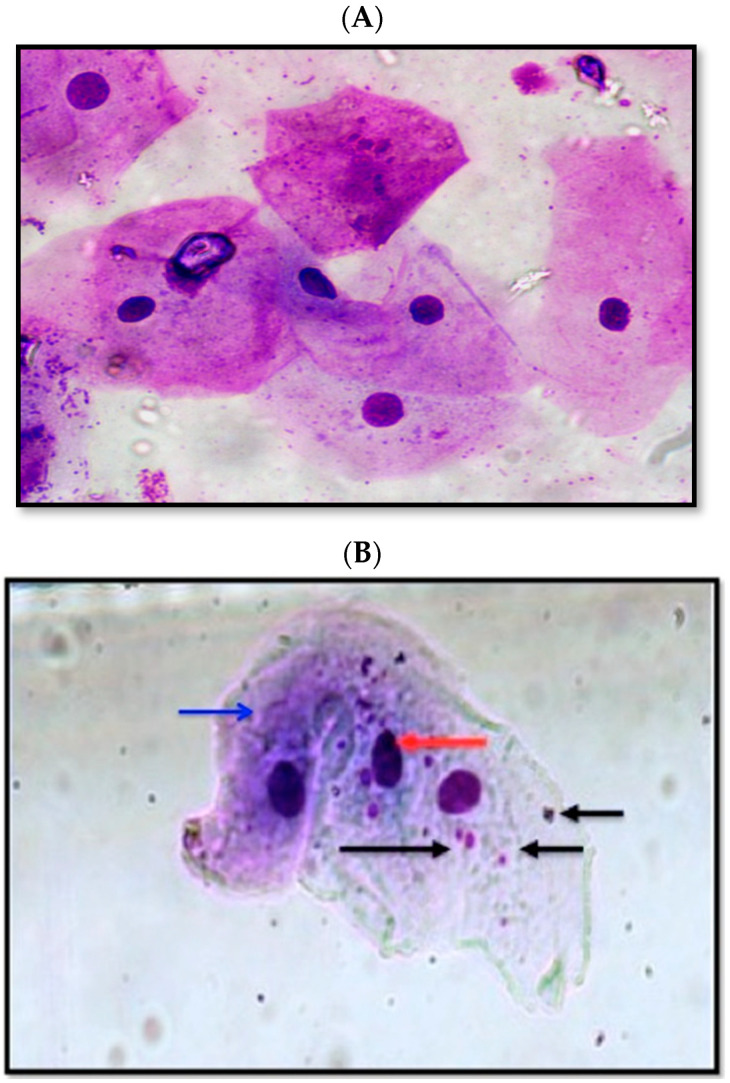
(**A**) Photomicrograph of non-snuff dipper buccal cell showing the nucleus (black arrow) and cytoplasm (red arrow) at 1000X and (**B**) photomicrograph of snuff-dipper buccal cell showing multiple micronuclei (black arrows), nucleus of buccal cells (red arrow), and cytoplasm (blue arrow) (Giemsa stain, 1000X).

The study presented mean values of 7.40 ± 3.83 pg/mL for TNF-α and 19.95 ± 12.36 micronuclei cell numbers in the study population. TNF-α levels among snuff users were 9.6 ± 3.3 pg/mL, which was significantly higher than levels in non-snuff users, 5.2 ± 3 pg/mL (*p* < 0.05) (Table 2). Similarly, the number of micronuclei among SD was 30.7 ± 7.8, which was a significantly higher than the number of micronuclei among NSD, 9.2 ± 3.3 (*p* < 0.05) (Table 2). Figure 1 presents the micronuclei observed in non-snuff dippers and snuff dippers.

The photomicrograph of NSD buccal cells is presented in Figure 1A. The cytoplasm and nucleus of cells in different sections was observed. The nuclei in the cells are denoted with a black arrow, and red arrows indicate cytoplasmic material in the cells, including water, protein, salts, and cellular components. Among the micrographs of buccal cells from snuff dippers (SD) (Figure 1B), in the micronuclei of damaged chromosome material expressed during cell division of a genetically unstable cell, cell death or cancer development was observed (black arrows) as extranuclear bodies. The cell nuclei (red arrow) and cytoplasm (blue arrow) among cells from SD were also observed (Figure 1B).

The study aimed to establish a correlation between age, duration of snuff use, TNF-α, and micronuclei (Table 2). Comparing snuff users (9.6 pg/mL; 30.7 average number of micronuclei) with non-snuff users (5.2 pg/mL; 9.2 average number of micronuclei), TNF-α and micronuclei showed a significant correlation (*p* ≤ 0.01). The levels of TNF-α were observed to be directly proportional to the micronuclei number. Thus, an increase in the TNF-α value simultaneously showed an increase in the micronuclei nuclei number, which indicates a positive correlation. 

In contrast, age did not show a significant correlation with TNF-α and micronuclei numbers (*p*-values, 0.454 and 0.099, respectively) (Table 3 and Table 4). However, the duration of snuff use was found to be positively correlated to TNF-α levels (*p* = 0.048) (Table 5) along with micronuclei numbers (*p* = 0.007) (Table 6). This positive correlation indicates that the prolonged use of snuff stimulates the TNF-α production over time and the rising levels of TNF-α possibly lead to overproduction of micronuclei.

## 4. Discussion

The present study aimed to determine the UWS TNF-α levels and cellular micronuclei numbers in snuff dippers (SD) compared with NSD (control subjects). The study showed that irrespective of age, the users (SD) displayed high levels of UWS TNF-α and micronuclei in the buccal vestibule. Moreover, the duration of use was found to be significantly associated with raised levels of TNF-α and cellular derangement overtime. Thus, the null hypothesis, that there is no difference in TNF-α and micronuclei numbers among SD and NSD, was rejected. 

Naswar is a combination of crushed tobacco leaves, slaked lime, and ashes, which is either inhaled or dipped in the vestibule of either side. After 5 min, the nicotine-associated products seep into the inner mucosa and exhibit a slight burning and numbing sensation [21]. The effect is mainly due to the high pH of un-ionized nicotine as well as tobacco-specific N-nitrosamines (TSNAs), which are carcinogenic and genotoxic. The literature reported a high level of incidence and toxic effects among males rather than females due to frequent exposure to narcotics. Similarly, the present study was inadvertently gender biased, as snuff dipping is commonly practiced among males with ages ranging from 18 to 62 years.

To date, biopsy and histopathological review are considered a gold standard practice; however, these tools are not sufficient in the screening of premalignant lesions and cancers. Each year, thousands of OSCC cases are reported at advanced stages despite the invention of advanced examination tools and technology [7]. Thus, late diagnosis leads to a high risk of morbidity and a low survival rate. Many authors suggested biomonitoring of these patients to detect pathological changes at an early stage of cancer for immediate preventive action [22,23]. Recent advances provide similar clinical evidence to use salivary biomarkers such as micronuclei and TNF-α as an initial assessment, as it is considered to be one of the most convenient and sensitive methods for genetic damage biocontrol [22]. Thus, the findings of the present study coincide with previous findings and prove that screening specific salivary biomarkers to categorize high-risk oral cancer patients can be considered an ideal method for early identification.

In the present research, a positive correlation with the duration of substance use was established with the TNF-α levels and micronuclei numbers. The literature suggests that the prolonged use of betel nuts and Naswar maintains consistent contact with the mucosa, resulting in slow release of nitrosamine [7]. Methylnitrosaminoproprionitrile was identified as the main component in smokeless tobacco that exhibits a carcinogenic effect [7]. Over time, the betel nut concentrates the squamous epithelial cells with nitrosamine, which is simultaneously activated by the P450 cytochrome [7]. Thus, the resulting intermediate products are unstable in nature and readily derange DNA replication, causing carcinogenesis. In contrast, the body’s immune system resists these effects and maintains cellular integrity due to the control of a tumor suppressor gene such as p53 [24,25,26]. However, the increased frequency of toxic agents impairs the tumor suppressor gene function, leading to loss of proliferative control. Therefore, the correlation of micronuclei with an increased duration of snuff use in the present study indicates a compromise in activity of the p53 tumor suppressor among the subjects.

However, the rising levels of micronuclei were identified to be dependent from TNF stimulation. According to previous studies, areca nuts have the potential to stimulate the production of TNF-α in saliva, which positively correlates with time and dose [1,27]. It was observed that prolonged use of snuff causes heavy tissue deposition and tenacious stains of the dipper site that stimulate inflammatory reactions, including platelet aggregation, increased fibrogenesis, phospholipase C activation, and mobilization of calcium ions as well as induced dysplastic changes [28]. Thus, continuous snuff use triggers chronic inflammation that leads to dedifferentiation of cells and hyperkeratosis in the vestibular dipping site. Consequently, the present outcomes coincide with previous findings and establish the positive influence of snuff duration on high levels of TNF-α levels. Micronuclei, as extranuclear cytoplasmic bodies induced by chromosomal aberration, are stimulated by alcohol, smoking tobacco, genotoxic agents, and chewing tobacco [29]. In the present study, SD cells showed significantly higher micronuclei presence compared to the NSD cells assessed. This increased induction of micronucleated cells in SD patients can be attributed to the genotoxic influence of organic solvents, aromatic hydrocarbons, and nitrosamine from the areca nut present in snuff. In a previous study by Chatterjee et al., it was revealed that micronucleus frequencies in cancer and precancerous cases were elevated 4-fold compared with significantly lower micronucleus levels in cells from non-malignant pathologies [30]. In a similar study by Amin et al., a direct relation of increasing micronuclei was presented from normal buccal cells to premalignant and malignant oral cells using microscopic evaluation [31]. Although these findings are similar to the present study, our study differs, as the oral cells were exposed to snuff (Naswar), which is a significantly new contribution to existing literature.

Outcomes of the present investigation showed that increasing age was not associated with an increase in TNF-α levels and micronuclei numbers. Bruunsgaard et al. [32] reported that increasing age is associated with a rise in inflammatory markers such as TNF-α, IL-6, cytokine antagonists, acute-phase proteins, and neopterin. Thus, iatrogenic inflammatory reactions in the elderly often reflect upon the age factor. Similarly, Orta et al. [33] observed that as a person ages, the function of the tissues and organs declines, which increases the susceptibility to DNA damage, resulting in genomic instability. Nevertheless, the impact of aging is still not elucidated, and the occurrence of DNA aberrations is inevitable due to the accumulation of reactive oxidative species over time. Thus, despite knowing that aging enhances inflammatory makers, the outcomes of the present study failed to comply with the findings of Bruunsgaard et al. [32], Milan- Mattos et al. [34], and de Gonzalo-Calvo et al. [35].

The present study proved the clinical significance of TNF-α and micronuclei in oral cancer screening of high-risk patients. Inflammatory mediators, including TNF-α, are induced in chronic inflammatory conditions such as periodontitis and gingivitis. Although periodontitis patients were excluded, the presence of plaque may have induced gingival inflammation, acting as a confounding factor for raised TNF-α levels [10]. In addition, the findings of the present study cannot be generalized, as the study sample size was limited, gender-biased (male dominant), and specific towards Pakistani snuff users. In addition, the presence of subclinical chronic diseases along with the type of collection technique (spitting/drooling) employed may influence salivary composition and biomarker levels [36,37]. Thus, future studies should be performed in accordance with a large sample size, equal gender distribution, and prospective cohort study design along with a proper detailed medical evaluation for the adjustment of confounding factors.

## 5. Conclusions

Snuff dippers showed significantly higher levels of USW salivary TNF-α and cellular micronuclei compared to non-snuff dippers (control subjects), irrespective of age. TNF-α and cellular micronuclei presented a positive correlation with the duration of snuff use. We conclude that TNF-α and micronuclei are potential salivary biomarkers for an oral biological effect of snuff (Naswar) users. Further investigations are recommended to validate the use of TNF-α and cellular micronuclei as salivary biomarkers for early diagnosis of oral squamous cell carcinoma.

## Figures and Tables

**Table 1:** General characteristic of the study groups.

Feature	Mean (SD)/(Frequency)
Age (years)	38.85 (±11.5)
Duration of snuff use (years)	20.43 (±12.7)
Site of dipping (*n* = 30) *	
Snuff use in lower vestibule	19 (63.3%)
Snuff use in upper vestibule	11 (36.6%)
Oral hygiene (*n* = 60) $	
Good	12 (20%)
Fair	40 (66.6%)
Poor	8 (13.3%)

* Snuff users (*n* = 30), $ snuff and non–snuff users.

**Table 2:** Comparison of TNF-α and micronuclei levels among study participants.

	Snuff Users	Non-Snuff Users	*p*-Value
Age (years)	41.9 (±12.2)	35.8 (±10.4)	0.84 ^§^
Duration of snuff (years)	20.43 (±12.79)	-	-
TNF-α (pg/mL)	9.6 (±3.3)	5.2 (±3.0)	0.01 ^§^
No of micronuclei (per 1000 cells counted)	30.7 (±7.8)	9.2 (±3.3)	0.008 ^§^

^§^*t*-test, *p*-value < 0.05 denotes statistical significance.

**Table 3:** Correlation between age and TNF-α levels.

		Age	TNF-α Levels
Age	Pearson correlation	1	0.099 *
	Sig. (2-tailed)		0.454
TNF-α levels	Pearson correlation	0.099 *	
	Sig. (2-tailed)	0.454	

* Correlation is not significant at the 0.099 level.

**Table 4:** Correlation between age and micronuclei score.

		Age	Micronuclei Score
Age	Pearson correlation	1	0.215 *
	Sig. (2-tailed)		0.099
Micronuclei score	Pearson correlation	0.215 *	
	Sig. (2-tailed)	0.099	

* Correlation is not significant at the 0.215 level.

**Table 5:** Correlation between duration of snuff use and TNF-α levels.

		Duration of Snuff Use	TNF-α Levels
Duration of snuff use	Pearson correlation	1	0.048 *
	Sig. (2-tailed)		0.801
TNF-α levels	Pearson correlation	0.048 *	
	Sig. (2-tailed)	0.801	

* Correlation is significant at the 0.048 level.

**Table 6:** Correlation between duration of snuff use and micronuclei score.

		Duration of Snuff Use	Micronuclei Score
Duration of snuff use	Pearson correlation	1	0.007 *
	Sig. (2-tailed)		0.973
Micronuclei score	Pearson correlation	0.007 *	
	Sig. (2-tailed)	0.973	

* Correlation is significant at the 0.007 level.

## Data Availability

Study Data can be accessed form the corresponding author on request.

## Appendix A

CPITN index: Community Periodontal Index was measured for assessment of periodontal status, by using a mouth mirror and a CPITN probe which was a specifically designed periodontal probe with a 0.5 mm ball tip and a black band between 3.5 and 5.5 mm and with rings at 8.5 and 11.5 mm from the ball tip. According to WHO protocol [13], the dentition was divided into 6 sextants which are defined by tooth numbers: 14–18, 13–23, 24–28, 38–34, 33–43, and 44–48, and it was coded as following: Code 0- healthy periodontium, Code 1—bleeding on gentle probing, Code 2—calculus deposits being felt on probing, Code 3—pocket 4–5 mm (black band on the probe partially visible), Code 4—pocket 6mm or more (black band on probe not visible) and Code x—excluded (less than two teeth present). The index teeth which have to be examined are 17, 16, 11, 26, 27, 37, 36, 31, 46, and 47. If no index teeth are present, all the remaining teeth in that sextant are examined and the highest score is recorded as the score for the sextant.
